# Clickable Vitamins as a New Tool to Track Vitamin A and Retinoic Acid in Immune Cells

**DOI:** 10.3389/fimmu.2021.671283

**Published:** 2021-07-08

**Authors:** Amelie V. Bos, Martje N. Erkelens, Sebastiaan T.A. Koenders, Mario van der Stelt, Marjolein van Egmond, Reina E. Mebius

**Affiliations:** ^1^ Department of Molecular Cell Biology and Immunology, Amsterdam University Medical Center, location VUmc, Amsterdam, Netherlands; ^2^ Department of Molecular Physiology, Leiden Institute of Chemistry, Leiden University, Leiden, Netherlands; ^3^ Department of Surgery, Amsterdam University Medical Center, location VUmc, Amsterdam, Netherlands

**Keywords:** copper-facilitated click chemistry, retinoid probes, retinoic acid, vitamin A metabolism, CD38

## Abstract

The vitamin A derivative, retinoid acid (RA) is key player in guiding adaptive mucosal immune responses. However, data on the uptake and metabolism of vitamin A within human immune cells has remained largely elusive because retinoids are small, lipophilic molecules which are difficult to detect. To overcome this problem and to be able to study the effect of vitamin A metabolism in human immune cell subsets, we have synthesized novel bio-orthogonal retinoid-based probes (clickable probes), which are structurally and functionally indistinguishable from vitamin A. The probes contain a functional group (an alkyne) to conjugate to a fluorogenic dye to monitor retinoid molecules in real-time in immune cells. We demonstrate, by using flow cytometry and microscopy, that multiple immune cells have the capacity to internalize retinoids to varying degrees, including human monocyte-derived dendritic cells (DCs) and naïve B lymphocytes. We observed that naïve B cells lack the enzymatic machinery to produce RA, but use exogenous retinoic acid to enhance CD38 expression. Furthermore, we showed that human DCs metabolize retinal into retinoic acid, which in co-culture with naïve B cells led to of the induction of CD38 expression. These data demonstrate that in humans, DCs can serve as an exogenous source of RA for naïve B cells. Taken together, through the use of clickable vitamins our data provide valuable insight in the mechanism of vitamin A metabolism and its importance for human adaptive immunity.

## Introduction

Numerous dietary studies have stressed the importance of vitamin A for proper functioning of the immune system as vitamin A insufficiency is correlated to multiple infectious diseases (1–3). Vitamin A, also known as retinol, needs to be metabolized into its active form retinoic acid (RA) to influence the immune system. Retinol is an essential fat-soluble molecule, which needs to be obtained *via* the diet. Dietary retinoids are absorbed by intestinal epithelial cells (IEC) and transported in chylomicrons, in the form of retinyl esters, *via* the lymphatic system and can be stored in the liver from where it can be transported to target cells (4). Vitamin A is converted into the metabolic active product RA within the IEC (5). First, retinol is converted into retinal under normal physiological conditions by microsomal retinol dehydrogenases (RDHs) in a reversible mechanism (6). Second, retinal is irreversibly converted by aldehyde dehydrogenases (ALDHs) to form RA (7). RA binds to nuclear receptors to regulate target gene expression.

Metabolized vitamin A has an extensive influence on the immune system. For instance, metabolism of vitamin A by epithelial cells is responsible for steering mucosal dendritic cells (DCs) towards CD103-expressing tolerogenic cells, which in turn gain ALDH enzyme activity to produce RA themselves (8–11). Moreover, metabolized vitamin A induces expression of gut homing molecules (11, 12), differentiation of T and B cells and antibody class switching (13–17). More specifically, mouse strains with lower ALDH enzyme activity secrete less immunoglobulin A (IgA) into the intestinal lumen, leading to higher levels of bacterial translocation into the intestinal lamina propria and mesenteric lymph nodes (MLN), compared to mice with high ALDH enzyme activity (18). While RA was shown to directly promote IgA class switching (17, 19), the lack of RA signalling in B cells abrogated antigen specific IgA responses after oral immunization and affected the microbial composition of the intestine (17). The source of RA for lymphocytes in intestinal tissues is not completely known, as dendritic cells (DCs), stromal cells, as well as epithelial cells have all been indicated as cells that express vitamin A converting enzymes (10, 20, 21). It is becoming clear that in mice mucosal DCs play a critical role in the vitamin A mediated effects for the immune system, thereby regulating mucosal adaptive immune responses (22). Murine experiments have demonstrated that DCs metabolize RA and skew B cell differentiation into IgA+ B cells (11, 18, 23). However, studies assessing the mechanistic contribution of dendritic cells to vitamin A metabolism and B cell functionality are exclusive to murine studies. Human studies have mainly demonstrated that human DCs can induce IL10-producing regulatory T cells (23–25) and induce the expression of gut homing receptor α4β7 (23, 26) in an RA-dependent mechanism.

To date, the uptake and metabolism of vitamin A within human immune cells has remained largely elusive. Partly because retinoids are small, lipophilic molecules which are difficult to detect *via* microscopy methods. Previously, we developed an activity-based probe with alkyne click handle to visualize and quantify ALDH activity (27). In line, we now designed, synthesized and applied novel bio-orthogonal retinoid-based probes (clickable probes), which were structurally and functionally, virtually indistinguishable from vitamin A. Yet they contain a functional group (an alkyne) to conjugate them to a fluorogenic dye to monitor retinoid molecules in real-time in immune cells, enabling their visualization by flow cytometry and microscopy. These clickable probes showed to be functionally comparable to their natural counterpart. The contribution of RA derived from dendritic cells to B cell functionality in humans has not been yet addressed. Using these probes we show that human B lymphocytes lack the machinery to metabolize retinol into RA, although they can internalize retinoids. Interestingly, exogenous RA directly CD38 expression on B cells. In contrast, human monocyte-derived dendritic cells (moDCs) have the machinery to metabolize retinol into RA. We observed that retinal, when metabolized by moDCs, led to increased CD38 expression on B cells, suggesting that human dendritic cells can influence B lymphocytes directly by the production of RA. Overall, this study shows that human dendritic cells can serve as a source of RA for B cells and that this influences B cell properties.

## Methods

### Synthesis of Clickable Retinoid Analogues

**Scheme 1:**
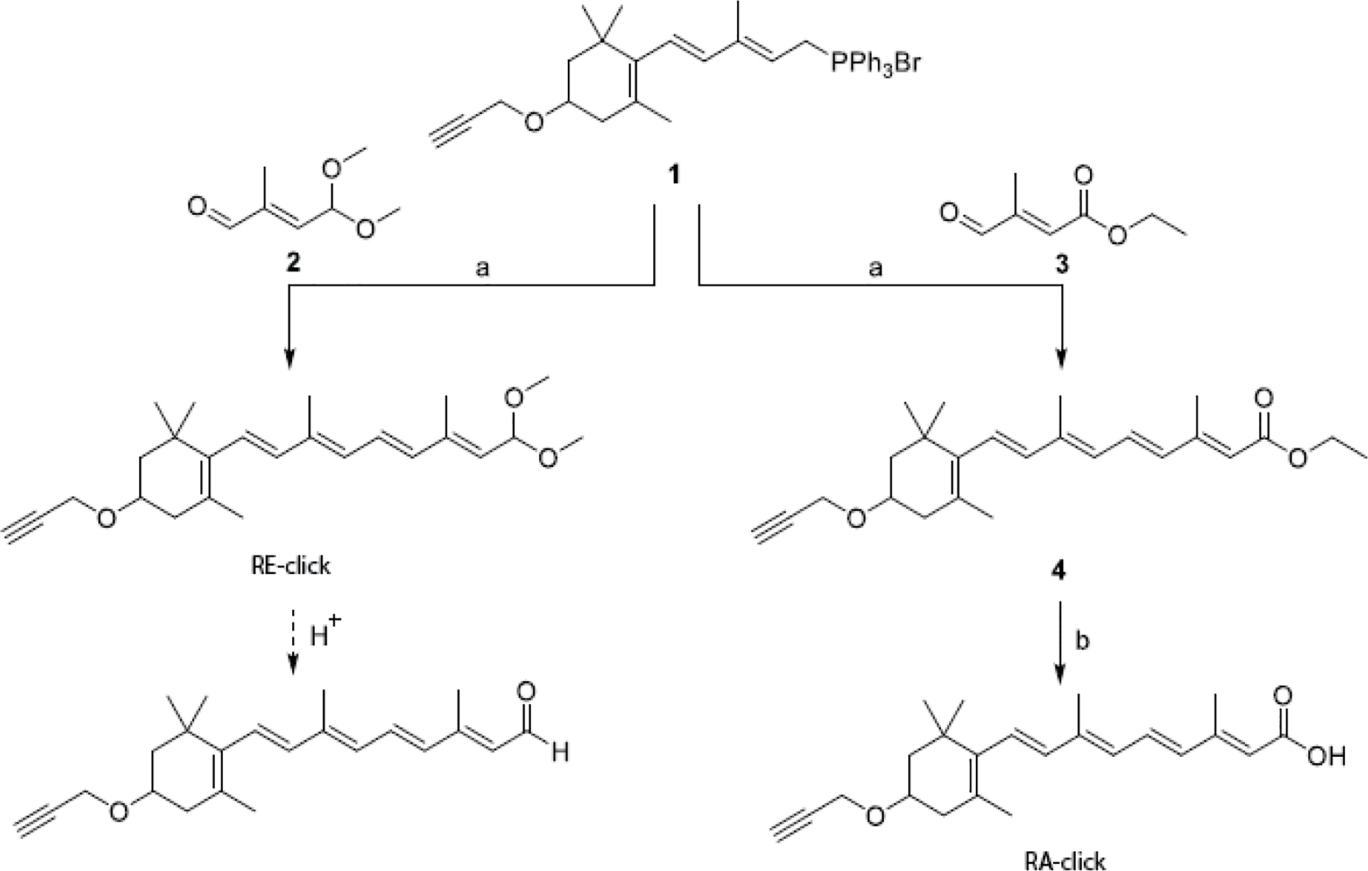
Synthesis of clickable vitamins. Reagents and conditions: a) *n*-Buli, THF, -78°C to RT, 3 h, 8% for RE-click and 39% for 4; b) 2M NaOH, MeOH, 50°C, 4 hours, 24%.

**Scheme 2:**
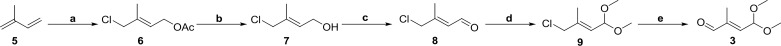
Synthesis of mono-protected aldehyde intermediate. Reagents and conditions: a) tBuOCl, AcOH, 1h and then CuSO_4_, H_2_SO_4_, 96h, 30%; b) Na_2_CO_3_, MeOH/H_2_O, 5h, 0°C, 33%; c) PCC, DCM, 90 min, 0°C, 51%; d) trimethyl orthoformate, pTsOH, MeOH, 24h, 67%; e) K_2_HPO_4_/KH_2_PO_4_, NaBr, DMSO, 24h, 80°C, 55%.

The clickable retinoid analogues were synthesized using the convergent synthesis route. Wittig salt (**1** in **scheme 1**) was coupled with aldehyde (**2** in **scheme 1**) to make the protected retinal analogue RE-click in 8% yield. Aldehyde (**2** in **scheme 1**) was synthesized following a literature procedure, which can be found in **scheme 2** (28). The poor yield is a result of several purification steps, which were required to separate the product from the unreacted aldehyde **2** and the facile deprotection of the acetal group under acidic conditions.

Retinal analogue RE-click was stored in ethanol at -80°C with the aldehyde still protected as dimethyl acetal. Exposure to slightly acidic conditions using a buffer or MilliQ instantly liberates the aldehyde, after which the compound can be used. The retinoic acid analogue was made using commercially available aldehyde (**3** in **scheme 1**), which resulted in compound **4 (4** in **scheme 1**) with a yield of 39%. This was then converted into the clickable retinoic acid analogue RA-click using 2M NaOH with a 24% yield, which was stored under the same conditions as the aldehyde analogue RE-click.

All reactions were performed using oven or flame‐dried glassware and dry solvents. Reagents were purchased from Sigma Aldrich, Acros, Biosolve, VWR, Fluka, Fischer Scientific and Merck and used as received unless stated otherwise. Tetrahydrofuran (THF) and *N,N*-dimethylformamide (DMF) were stored over molecular sieves before use. All moisture sensitive reactions were performed under a nitrogen atmosphere. TLC analysis was performed using Merck aluminum sheets (TLC silica gel 60/Kieselguhr F_254_. Compounds were visualized using a solution of KMnO_4_ (7.5 g), K_2_CO_3_ (50 g), 10% NaOH (6 mL) in H_2_O (1L). Column chromatography was performed using Screening Device B.V. silica gel (particle size of 40 – 63 µm, pore diameter of 60 Å) with the indicated eluents. ^1^H‐ and ^13^C‐NMR spectra were recorded on Brüker AV-400 (400 MHz and 101 MHz, respectively) or Brüker AV-500 MHz (500 MHz and 150 MHz, respectively) using CDCl_3_ as solvent. Chemical shifts are reported in ppm (δ) relative to the residual solvent peak or tetramethylsilane. Coupling constants are given in Hz. High‐resolution mass spectometry (HRMS) analysis was performed with a LTQ Orbitrap mass spectrometer (Thermo Finnigan), equipped with an electronspray ion source in positive mode (source voltage 3.5 kV, sheath gas flow 10 mL/min, capillary temperature 250°C) with resolution R = 60000 at m/z 400 (mass range m/z = 150 – 2000) and dioctyl phthalate (m/z = 391.28428) as a “lock mass”, or with a Synapt G2-Si (Waters), equipped with an electronspray ion source in positive mode (ESI-TOF), injection *via* NanoEquity system (Waters), with LeuEnk (m/z = 556.2771) as “lock mass”. Eluents used: MeCN:H_2_O (1:1 v/v) supplemented with 0.1% formic acid. The high-resolution mass spectrometers were calibrated prior to measurements with a calibration mixture (Thermo Finnigan). The retinoids were handled under dark conditions using amber colored flasks or aluminum foil when containing more than 3 conjugated double bonds. Retinoid intermediates were stored in the dark under nitrogen at -30°C and final compounds as powder or as EtOH stock at -80°C under nitrogen atmosphere. For further information on retinoid handling and storage we refer to the review from Barua and Furr (29).

### (E)-4-Chloro-3-methylbut-2-en-1-yl acetate (6):

To a stirred solution of isoprene **5** (**5** in **scheme 2**) (20 mL, 0.20 mol) in acetic acid (60 mL) at 0°C under N_2_ was added tert-butyl hypochlorite (18 mL, 0.16 mol). The reaction mixture was stirred for 1 hour and then quenched with H_2_O and extracted with Et_2_O. The organic layer was washed with NaHCO_3_ sat. aq. and brine, dried with MgSO_4_, filtered and concentrated under reduced pressure. The residue was then dissolved in acetic acid (60 mL) and to this was added CuSO_4_ (320 mg, 2.0 mmol) and H_2_SO_4_ (0.2 mL, 4 mmol). The reaction mixture was stirred for 96 hours at room temperature and was then quenched with H_2_O and extracted with Et_2_O. The organic layer was washed with NaHCO_3_ sat. aq. and brine, dried with MgSO_4_, filtered and concentrated under reduced pressure. Purification of the residue by column chromatography afforded the title compound **6** (**6** in **scheme 2**) (7.8 g, 48 mmol, 30%). ^1^H NMR (400 MHz, CDCl_3_) δ 5.70 (t, J = 6.8 Hz, 1H), 4.62 (d, J = 6.9 Hz, 2H), 4.02 (s, 2H), 2.06 (s, 3H), 1.82 (s, 3H). ^13^C NMR (101 MHz, CDCl_3_) δ 170.4, 136.6, 123.5, 60.4, 50.5, 20.5, 14.2. Spectroscopic data for H-NMR are in agreement with those reported (30).

### (E)-4-Chloro-3-methylbut-2-en-1-ol (7):

Compound **6** (7.8 g, 48 mmol) was dissolved in MeOH (120 mL). To this was added a solution of Na_2_CO_3_ (7.6 g, 72 mmol) in H_2_O (40 mL). The reaction mixture was stirred for 5 hours at 0°C, filtered and concentrated under reduced pressure. The remaining water layer was then extracted with CHCl_3_. The organic layer was washed with brine, dried with MgSO_4_, filtered and concentrated under reduced pressure. Purification of the residue by column chromatography (Pent/Et_2_O) afforded the title compound **7** (1.9** g**, 16 mmol, 33%). R_f_ (50% Et_2_O in Pent) = 0.5. ^1^H NMR (400 MHz, CDCl_3_) δ 5.70 (s, 1H), 4.15 (s, 2H), 4.02 (s, 2H), 3.43 (s, 1H), 1.76 (s, 3H). ^13^C NMR (101 MHz, CDCl_3_) δ 128.8, 58.5, 51.2, 42.7, 14.1.

### (E/Z)-4-Chloro-3-methylbut-2-enal (8):

A solution of **7** (1.9 g, 16 mmol) in dry DCM (20 mL) was added to a stirred suspension of PCC (5.1 g, 24 mmol) in dry DCM (40 mL) at 0°C under N_2_ containing molecular sieves. The reaction mixture was stirred for 90 minutes at 0°C and was then filtered over celite and concentrated under reduced pressure. Purification of the residue by column chromatography (Et_2_O/Pent) afforded the title compound **8** (**8** in **scheme 2**) (0.94 g, 8.0 mmol, 51%; 1:0.3 E/Z mixture). R_f_ (50% Et_2_O in Pent) = 0.8. ^1^H NMR (400 MHz, CDCl_3_) δ 10.04 (d, J = 7.7 Hz, 1H), 6.12 (d, J = 7.7 Hz, 1H), 4.12 (s, 2H), 2.28 (s, 3H); ^13^C NMR (101 MHz, CDCl_3_) δ 191.0, 155.2, 128.6, 49.3, 15.5.

### (E)-4-Chloro-1,1-dimethoxy-3-methylbut-2-ene (9):

To a solution of **8** (**8** in **scheme 2**) (0.94 g, 7.9 mmol) in MeOH (7 mL) was added trimethyl orthoformate (7.1 mL, 40 mmol) and PTSA (0.80 mmol, 84 mg). The reaction mixture was stirred overnight at room temperature and was then quenched with NaHCO_3_ aq. and extracted with Et_2_O. The organic layer was washed with brine, dried with MgSO_4_, filtered and concentrated under reduced pressure. Purification of the residue by column chromatography (Et_2_O/Pent) afforded the title compound **9** (**9** in **scheme 2**) (0.88 g, 5.3 mmol, 67%). R_f_ (10% Et_2_O in Pent) = 0.5. ^1^H NMR (400 MHz, Chloroform-d) δ 5.51 (d, 6.0 Hz, 1H), 5.04 (d, J = 6.2 Hz, 1H), 4.01 (s, 2H), 3.32 (s, 6H), 1.85 (s, 3H). ^13^C NMR (101 MHz, CDCl_3_) δ 137.4, 126.6, 99.6, 52.2, 50.6, 14.9.

### (E)-4,4-Dimethoxy-2-methylbut-2-enal (2):

To a stirred solution of **9** (**9** in **scheme 2**) (0.78 g. 4.7 mmol) in DMSO (25 mL) was added K_2_HPO_4_ (1.1 g, 6.2 mmol), KH_2_PO_4_ (0.23 g, 1.7 mmol) and NaBr (73 mg, 0.71 mmol). The mixture was stirred for 18 hours at 80°C. Then H_2_O was added and the mixture extracted with CHCl_3_. The organic layer was washed with brine, dried with MgSO_4_, filtered and concentrated under reduced pressure. Purification by column chromatography (Et_2_O/Pent) afforded the title compound **2** (0.39 g, 2.7 mmol, 57%). R_f_ (10% Et2O in Pent) = 0.3. ^1^H NMR (400 MHz, CDCl_3_) δ 9.49 (s, 1H), 6.39 (d, J = 5.9 Hz, 1H), 5.27 (d, J = 5.9 Hz, 1H), 3.38 (s, 6H), 1.85 (s, 3H); ^13^C NMR (101 MHz, CDCl_3_) δ 194.4, 146.7, 141.5, 99.1, 52.5, 9.5. Spectroscopic data are in agreement with those reported (28).

### 2-((1E,3E,5E,7E)-9,9-Dimethoxy-3,7-dimethylnona-1,3,5,7-tetraen-1-yl)-1,3,3-trimethyl-5-(prop-2-yn-1-yloxy)cyclohex-1-ene (RE-click):

To a stirred solution of **1** (**1** in **scheme 1**) (420 mg, 0.70 mmol) in dry THF (5 mL) at -78° under N_2_ was added *n*-BuLi (0.3 mL, 2.5M in hexane, 0.76 mmol). The mixture was stirred for 30 minutes at -78°C and then a solution of **2** (**2** in **scheme 1**) (100 mg, 0.70 mmol) in dry THF (5 mL) was added. The reaction mixture was stirred for 1 hour at -78°C and then for 3 hours at room temperature. Then the reaction was quenched with NH_4_Cl aq., extracted with Et_2_O. The combined organic layers were washed with H_2_O and brine, dried with MgSO_4_, filtered and concentrated under reduced pressure. Purification of the residue by column chromatography (Et_2_O/Pent) with neutral silica afforded the title compound RE-click (21 mg, 55 μmol, 7.9%). ^1^H NMR (500 MHz, CDCl_3_) δ 6.70 – 6.07 (m, 5H), 5.56 – 5.13 (m, 2H), 4.22 (d, *J* = 2.4 Hz, 2H), 3.86 (dtt, *J* = 12.0, 5.7, 3.3 Hz, 1H), 3.33 (d, *J* = 10.1 Hz, 6H), 2.46 – 2.38 (m, 2H), 2.07 (dd, *J* = 16.8, 9.6 Hz, 1H), 1.96 (s, 3H), 1.91 (s, 3H), 1.86 – 1.82 (m, 1H), 1.73 (s, 3H), 1.44 (t, *J* = 12.0 Hz, 1H), 1.07 (s, 6H). ^13^C NMR (126 MHz, CDCl_3_) δ 154.6, 138.9, 138.3, 137.7, 136.3, 136.1, 130.4, 127.8, 126.1, 125.9, 125.7, 100.2, 80.4, 73.8, 71.6, 55.1, 52.2, 44.5, 39.2, 36.8, 30.2, 28.6, 21.6, 13.2, 12.7. HRMS (ESI) m/z: [M + H]^+^ calculated for deprotected RE-click C_23_H_30_O_2_: 339.23186, found: 339.23185.

### Ethyl (2E,4E/Z,6E,8E)-3,7-Dimethyl-9-(2,6,6-trimethyl-4-(prop-2-yn-1-yloxy)cyclohex-1-en-1-yl)nona-2,4,6,8-tetraenoate (4):

To a stirred solution of **1** (**1** in **scheme 1**) (0.50 g, 0.83 mmol) in dry THF (4 mL) at -78° under N_2_ was added *n*-BuLi (0.50 mL, 1.6M in hexane, 0.83 mmol). The mixture was stirred for 30 minutes at -78°C and then ethyl 3-methyl-4-oxocrotonate **3** (0.10 mL, 0.76 mmol) was added. The reaction was stirred for 1 hour at -78°C and then for 2 hours at room temperature. Then the reaction was quenched with NH_4_Cl aq. And extracted with Et_2_O. The combined organic layers were washed with H_2_O and brine, dried with MgSO_4_ and concentrated under reduced pressure. Purification of the residue by column chromatography (EtOAC/Pent) afforded the title compound **4** (**4** in **scheme 1**) (0.11 g, 0.30 mmol, 39%) as a yellow oil as an E/Z mixture (1:0.3). R_f_ (10% EtOAc in Pent) = 0.95. NMR spectra are obtained from the mixture of stereoisomers. ^1^H NMR (400 MHz, CDCl_3_) δ 6.98 (dd, *J* = 15.1, 11.4 Hz, 1H), 6.55 – 6.09 (m, 4H), 5.93 – 5.76 (m, 1H), 4.25 – 4.21 (m, 2H), 4.21 – 4.13 (m, 2H), 3.92 – 3.81 (m, 1H), 2.48 – 2.38 (m, 2H), 2.37 – 2.32 (m, 3H), 2.12 – 2.03 (m, 1H), 2.01 – 1.94 (m, 3H), 1.88 – 1.82 (m, 1H), 1.73 (s, 3H), 1.45 (t, *J* = 12.0 Hz, 1H), 1.29 (t, *J* = 7.1 Hz, 3H), 1.11 – 1.05 (m, 6H). ^13^C NMR (101 MHz, CDCl_3_) ^13^C NMR (101 MHz, CDCl_3_) δ 167.13, 152.54, 139.10, 137.98, 137.59, 135.51, 130.70, 129.95, 127.48, 126.60, 118.79, 80.30, 73.88, 71.48, 59.64, 55.13, 44.39, 39.23, 36.80, 30.16, 28.58, 21.62, 14.32, 13.78, 12.87. HRMS (ESI) m/z: [M + H]^+^ calculated for C_25_H_34_O_3_: 383.25807, found 383. 25801.

### (2E,4E/Z,6E,8E)-3,7-Dimethyl-9-(2,6,6-trimethyl-4-(prop-2-yn-1-yloxy)cyclohex-1-en-1-yl)nona-2,4,6,8-tetraenoic acid (RA-click):

To a stirred solution of **4** (**4** in **scheme 1**) (64 mg, 0.167 mmol) in MeOH (2.5 mL) was added 2M NaOH (aq) (2.5 mL). The reaction was heated at 50°C for 3 hours and then another 5 mmol of NaOH (200 mg) was added. The reaction was then stirred for 1 hour at 50°C and then made acidic with 1M HCl. The product was then extracted with EtOAc, washed with brine, dried over MgSO4, filtrated and concentrated. Purification by column chromatography (Et_2_O/Pent) afforded the title compound RA-click (14 mg, 39 μmol, 24%; 7:3 4E/Z). The E/Z mixture was inseparable and prone to further isomerization under the influence of light. As retinoids are rapidly converted *in vivo* into their biological equilibrium of stereoisomers. RA-click was used as the reported E/Z mixture.^21,22^ NMR spectra are obtained from the mixture of stereoisomers. ^1^H NMR (500 MHz, CDCl_3_) δ 7.04 (dd, *J* = 15.0, 11.4 Hz, 1H), 6.62 – 6.50 (m, 1H), 6.35 – 6.11 (m, 3H), 5.94 – 5.79 (m, 1H), 4.22 (s, 2H), 3.91 – 3.83 (m, 1H), 2.47 – 2.40 (m, 2H), 2.39 – 2.33 (m, 3H), 2.13 – 2.04 (m, 1H), 2.02 – 1.96 (m, 3H), 1.88 – 1.83 (m, 1H), 1.74 (s, 3H), 1.48 – 1.42 (m, 1H), 1.08 (s, 6H). ^13^C NMR (126 MHz, CDCl_3_) δ 172.1, 155.1, 139.8, 137.9, 137.6, 135.2, 131.7, 129.9, 127.9, 126.8, 117.8, 80.3, 73.9, 71.5, 55.2, 44.4, 39.2, 36.8, 30.2, 28.6, 21.6, 14.0, 12.9. LC-MS (ESI) m/z: [M + H]^+^ calculated for C_23_H_30_O_3_: 355.22677, found: 355.16667.

### Human Primary Cells

Blood samples were collected from donors after they had given informed consent, in accordance with the guidelines of the Medical Ethical Committee of the VU University Medical Center (The Netherlands), and the declaration of Helsinki. Blood was used to isolate peripheral blood mononuclear cell (PBMCs) using Lymphoprep (Axis-Shield) density gradient centrifugation.

### B Cell Cultures

B lymphocytes were purified from the PBMC fraction using negative isolation EasySep™ Human Naïve B Cell Isolation Kit or EasySep™ Human Pan-B Cell Enrichment Kit (both Stemcell Technologies). B cells were cultured in RPMI supplemented with 10% FCS, 1%PSG (both Thermo Scientific), 1 µg/ml anti-IgM (Clone DA4-4, MyBiosource), 20 ng/ml IL-4 (Immunotools) and 1 µg/ml anti-CD40 antibody (Clone G28.5 BioXcell) for 7 days. When indicated, 1 µM retinoic acid, retinal, RA-click or RE-click was added once at day 0.

### Dendritic Cell Culture

CD14^+^ monocytes were isolated from the PBMC fraction with positive selection using CD14 magnetic microbeads (Miltenyi Biotec) and cultured in RPMI supplemented with 10% FCS, 1%PSG (all Thermo Scientific), 20 ng/ml GM-CSF and 20 ng/ml IL-4 (both Immunotools) for 7 days in order to induce the development of dendritic cells. When indicated 1 µM retinoic acid, RE-click or RA-click was added once at day 0.

### Co-Culture of B Cells and Dendritic Cells

Naïve B cells were labelled with Cell trace Violet (ThermoFisher) and seeded in RPMI containing 1%PSG (Thermo Scientific), N21-MAX Vitamin A Free Media Supplement (R&D systems) and B cell stimulating factors as described above (anti-IgM, IL-4 and anti-CD40 antibody). At a ratio of 1:1 differentiated moDCs were added. Co-cultures were carried out for 9 days in the presence of 250 nM all-trans retinal, 250 nM all-trans retinoic acid or ethanol as a vehicle control.

### Aldefluor Assay

Aldefluor assay (Stemcell Technologies) was performed according to manufacturer’s protocol.

### Incubation of Cells With Clicklable Retionoids and Click Chemistry

In all experiments, cells were incubated with 1 µM clickable retinoids for 1 hour at 37°C in RPMI medium, without serum, unless otherwise indicated. Cells were fixed using 1% PFA for 30 minutes on ice. 5 µl (100 mM) CuSO4 and 5 µl (1 M) NaAsc (both Sigma Aldrich) we mixed and vortexed for a few minutes until the colour turned from brown to yellow. 2,5 µl THPTA (100 mM) (Sigma Aldrich) was added to this mixture, together with 487.5 µl PBS. After a few minutes, 0,5 µl commercially available azide labelled with alexa fluor 647/488 (ClickChemistryTools) was added and incubated for 3 additional minutes. This is called the ‘click mixture’ and is used for incubation with target cells, in order to visualize clickable retinoids. The azide and alkyne form a triazole, *via* a copper(I)-catalyzed [2 + 3] cyclo-addition. The interaction between the azide group and alkyne is covalent, resulting in a stable product. Once the interaction is formed with the retinoid probe, the fluorophore cannot leave anymore. Per click condition, around 100 000 cells were treated with 100 ul click mixture for 1 hour at 37°C. Subsequently, cells were washed with 2 mM EDTA, 3% BSA and finally PBS using the centrifuge at 1500 rpm at 4°C.

### Microscopy

Upon clicking the probes with a fluorophore, cells were treated with DAPI (Thermo Scientific). Single cells were embedded in Mowiol (Sigma Aldrich) on a microscope slide and covered with a coverslip. Slides were imaged using a DM6000 fluorescent microscope (Leica) or a SP8 confocal microscope (Leica).

### Flow Cytometry

B lymphocytes were stained with anti-CD38 APC (Clone HIT2, Biolegend) and anti-IgA biotin (Novex). After washing, streptavidin alexa-488 and fixable viability dye eFluor780 (eBioscience) were used. Differentiated monocyte-derived dendritic cells were incubated with an CD103 APC (Clone Ber-ACT8, BD Bioscience) and fixable viability dye eFluor780. Samples were acquired on LSR-Fortessa X20 (BD Bioscience) and data was analyzed with Flowjo software (Tree Star). Data represented in histograms are normalized to mode, meaning data is normalized to the highest value per condition.

## Result

### Characterization of Clickable Retinoids Within Human Dendritic Cells

**Figure 1:**
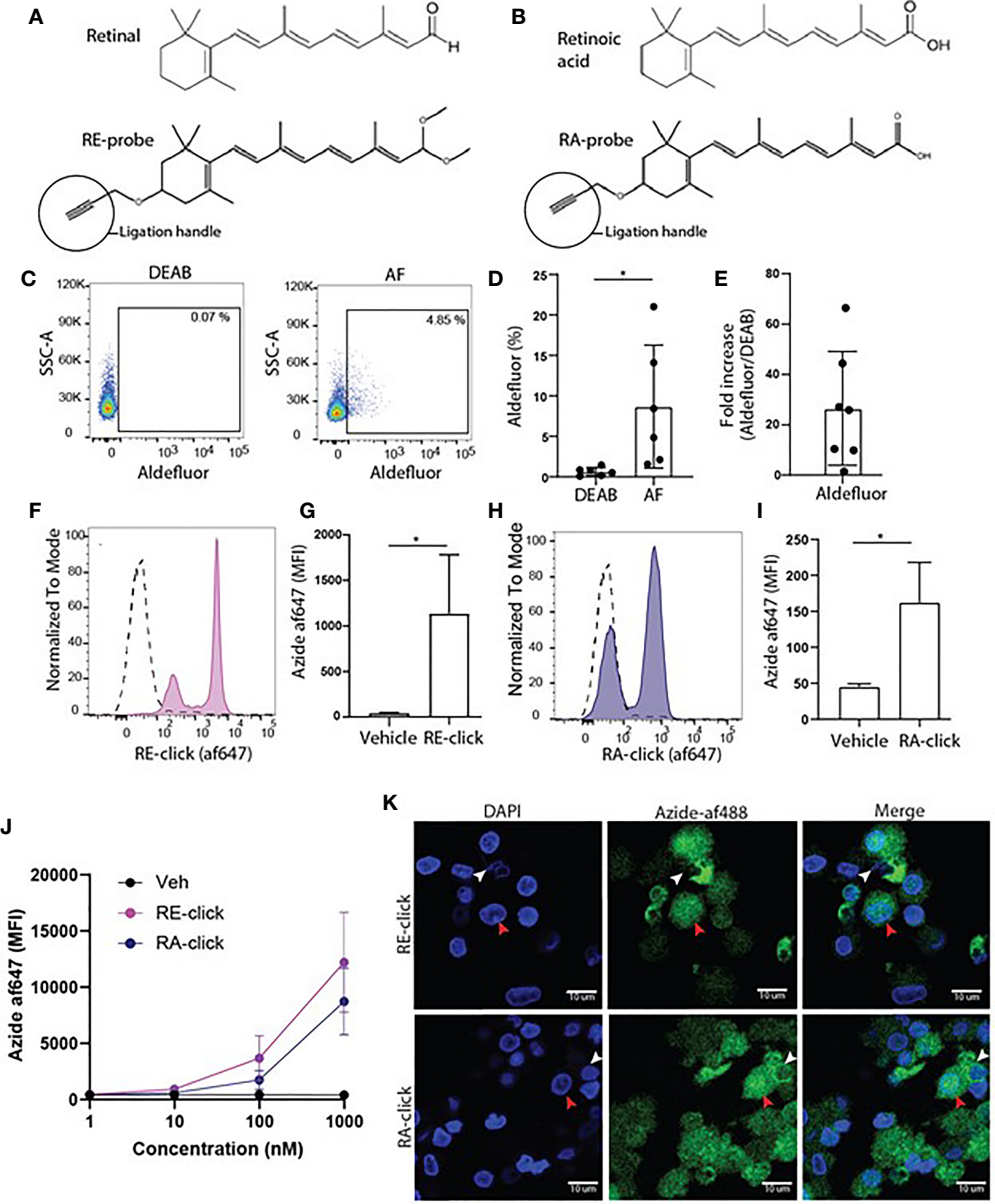
Monocyte-derived dendritic cells have the capacity to take up and metabolize retinoids. **(A)** Chemical structure of retinal and its synthetic clickable counterpart which contains an alkyne ligation group to allow visualization. **(B)** Chemical structure of retinoic acid and its synthetic clickable couterpart which contains an alkyne ligation group to allow visualization. **(C–K)** Monocytes were isolated from blood and differentiated into monocyte-derived dendritic cells (moDCs) during 7 days of culture in the presence of GM-CSF and IL-4. **(C)** Representative dot plots of aldeluor positivity in moDCs and DEAB-treated negative control. **(D)** Quantification of the aldefluor positivity in moDCs from 6 different donors. **(E)** Quantification of the increase of aldefluor positivity in moDCs compared to the paired DEAB control from 7 different donors. **(F–K)** Clickable retinoid probes were incubated for one hour in RPMI at 37°C with differentiated moDCs. **(F)** Representative histogram of the uptake of RE-click (pink) compared to the vehicle control (dotted line) in moDCs. **(G)** Quantification of the uptake RE-click compared to the vehicle control in moDCs from 3 different donors. **(H)** Representative histogram of the uptake of RA-click (blue) compared to the vehicle control (dotted line) in moDCs. **(I)** Quantification of the uptake of RA-click compared to the vehicle control in moDCs from 4 different donors. **(J)** Differentiated moDCs were incubated with multiple concentrations of RE­ and RA-click ranging from 1000 to 1 nM. Quantification of the uptake of the clickable probes was detemtined using flow cytometry using blood form 3 different donors **(K)** moDCs were put on a coverslip to assess the spatial distribution of retinoid probes (green) and nuclei (DAPI, blue) using confocal microscopy. White arrow indicates cytoplasmic accumulation, whereas the red arrow indicates nuclear localization. Data are presented as mean± SD. Student's t test; *p < 0.05.

To study the cellular uptake of endogenous vitamin A metabolites, clickable retinal (RE-click) and retinoic acid (RA-click) were designed to resemble its natural counterparts, including an additional alkyne ligation handle to conjugate them to a fluorescent dye, *via* an azyde, to allow their detection in cells using microscopy or flow cytometry (**Figures 1A, B**). First, we used all white blood cells to get an insight into the uptake of the RE- and RA-clickable probes within peripheral immune subsets. Briefly, cells were placed in RPMI without any further addition at 37°C for 1 hr and the probes were added to the medium to allow cellular uptake. For detection of the probes within cells fluorescently tagged azide was ligated to the alkyne group present on the probe *via* a copper-catalysed reaction. Using flow cytometry, uptake of the clickable probes in granulocytes, monocytes and lymphocytes was assessed by gating on their differences in flow cytometric characteristics for forward- and side-scatter (**Supplemental Figure 1A**). To determine background signal click chemistry was performed on cells receiving vehicle instead of the clickable probes. It was observed that lymphocytes, monocytes and granulocytes all had the capacity to take up the probes, although to varying degrees (**Supplemental Figures 1B–G**).

Vitamin A metabolism plays a prominent role in intestinal DCs. Therefore, human DCs were used to assess the applicability of the clickable retinoids. We first verified whether human monocyte-derived dendritic cells (moDCs) showed ALDH activity as measured with the aldefluor assay. This assay utilizes a fluorescent substrate, which is converted by ALDH enzymes, leading to the accumulation of fluorescent signal within the cells that express the enzymes. The amount of fluorescent product is proportional to the ALDH activity in the cells. As a negative control, diethylaminobenzaldehyde (DEAB) reagent is used to inhibit ALDH activity, which sets the threshold for background signal. Human moDCs showed enhanced aldefluor activity ranging from 2 to 21% within donors compared to 0.6 ± 0.5% SD in DEAB control samples (**Figures 1C, D**). This suggests that moDCs have ALDH enzyme activity, which varied per donor. The average fold increase in aldefluor positivity compared to the DEAB control was twenty-seven (**Figure 1E**). Subsequently, MoDCs were incubated with the clickable probes to assess whether they were capable of taking up retinoids. We observed that all moDCs demonstrated enhanced mean fluorescent intensity (MFI) for the RE-click compared to the vehicle treated negative control, resulting in two separate peaks within the moDCs (**Figures 1F, G**). The RA-click was taken up by approximately half of the moDCs, as the other half of the cells were negative for the probe (**Figures 1H, I**). Next, we titrated the uptake of our probes in concentrations ranging from 1- 1000 nM during a 1 hr incubation at 37°C in RPMI to see at which dose they were still detectable within cells using flow cytometry. We observed that RE-click and RA-click could still be detected at concentrations ranging from 100 to 1000 nM compared to vehicle control, while below 10 nM uptake was not observed above the vehicle control (**Figure 1J**). To subsequently visualize the spatial distribution of the probes within the cells, moDCs were incubated with the clickable probes for 1 hr at 37°C in RPMI and analysed using microscopy. In line with flow cytometric data, enhanced MFI compared to vehicle was seen after incubation with clickable retinoid probes (**Supplemental Figure 2**). The RE-click mainly accumulated outside the nucleus of moDCs in the cytosol, whereas the RA-click was also detected within the nucleus in a small fraction of the cells (**Figure 1J**). Three dimensional analysis was performed and demonstrated that both retinoid probes crossed the cell membrane and were internalized (**Supplemental Videos 1** and **2**). Taken together our data demonstrates that the clickable probes are a helpful tool to study retinoid uptake, demonstrating that human moDCs have the capacity to internalize retinoids.

### Uptake of Clickable Retinoids in Human Dendritic Cells Is Independent of Protein and Temperature

**Figure 2:**
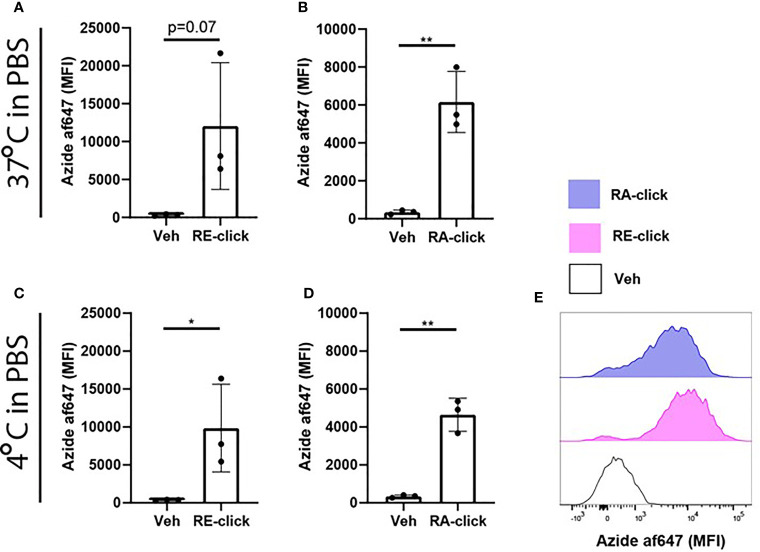
Uptake of dickable retinoids in human dendritic cells is independent of protein and temperature. Monocytes were isolated from blood and differentiated into monocyte-derived dendritic cells (moDCs) during 7 days of culture in the presence of GM-CSF and IL-4 from 3 different donors. Uptake of **(A)** RE-click and **(B)** RA-dick compared to the vehicle control in differentiated moDCs after incubation in phosphate buffered saline (PBS) for one hour at 37°c was determined by flowcytometric analysis and expressed as mean fluorescent intensity (MFI). Uptake of **(C)** RE-click and **(D)** RA-click compared to the vehicle control in differentiated moDCs after incubated in phosphate buffered saline (PBS) for one hour at 4°C was determined by flowcytometric analysis and expressed as MFI. **(E)** Representative histogram of uptake of RE- (pink) and RA-click (blue) compared to the vehicle control (solid line) in moDCs at 4°C, as shown in **(C, D)** Data are presented as mean± SD. Student's t test; *p < 0.05, **p < 0.01.

Since we demonstrated that the clickable retinoids can be used to quantify cellular uptake within human moDCs, we utilized the probes to study the mechanism of uptake into more detail. For blood-borne transport retinol is bound to retinol binding protein-4 (RBP4) (31). Target cells expressing receptors to recognize this retinoid-protein complex can take up retinol, thereby providing the cells with cytoplasmic retinol for metabolism (31). Therefore, we questioned whether the uptake of our probes requires carrier proteins. Hereto we incubated moDCs with the RE- and RA-click in phosphate buffered saline (PBS) without any further supplementation. Using flow cytometry, we observed that moDCs showed enhanced MFI for both the RE- and RA-click compared to the vehicle control (**Figures 2A, B**), suggesting that uptake can be achieved in the absence of proteins. Moreover, multiple studies have proposed that the uptake of free retinoids happens *via* passive diffusion (32–34). To assess whether uptake of clickable retinoids is dependent on ATP-driven metabolism, we incubated moDCs with our probes at 4°C. It was observed that both RE- and RA-click were taken up by moDCs when incubated at 4°C (**Figures 2C, D**). Although this suggests that the uptake of clickable probes is ATP-independent, we did observe that a fraction of the moDCs had not taken up any clickable probe, indicative of uptake by a subset of DCs(**Figure 2E**). Taken together, the clickable probes can be used in a straightforward method to study the cellular uptake of retinoids in immune cells.

### Clickable Retinoic Acid Is Functionally Active Within Human Dendritic Cells

**Figure 3:**
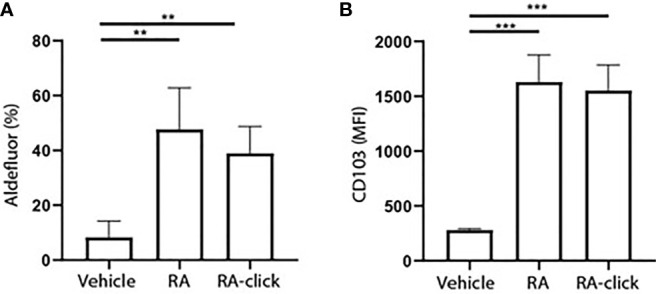
Clickable RA probe induces tolerogenic phenotype in monocyte-derived dendritic cells. Monocytes were isolated from blood and differentiated into monocyte-derived dendritic cells (moDCs) during 7 days of culture in the presence of GM-CSF and IL-4 from 3 different donors. **(A)** Aldefluor positivity and **(B)** CD103 expression in moDCs differentiated in the presence of natural RA or RA­ click. Data are presented as mean± SD. Student's t test; ANOYA; **p < 0.01, ***p < 0.001.

Subsequently, the functionality of RA-click on human DC behaviour was examined. Others have demonstrates that RA polarizes peripheral monocytes into CD103^+^ tolerogenic DCs with high ALDH activity (23, 26). Similarly, we differentiated monocytes for 7 days within the presence of RA-click. Retinal was excluded in these assays, as it has no functional effect on moDC differentiation. In the presence of natural RA, and likewise the RA-click, ALDH activity increased in moDCs (**Figure 3A**). Moreover, the expression of CD103 increased compared to conventional cultured moDCs after incubation with natural RA and also upon incubation with RA-click (**Figure 3B**). Together, our data demonstrates that the RA-click can induce the differentiation of moDC in a similar fashion as its natural counterpart, suggesting the probe is also suitable for functional assays.

### Naïve B Cells Take Up Exogenous Retinoids but Cannot Produce Retinoid Acid

**Figure 4:**
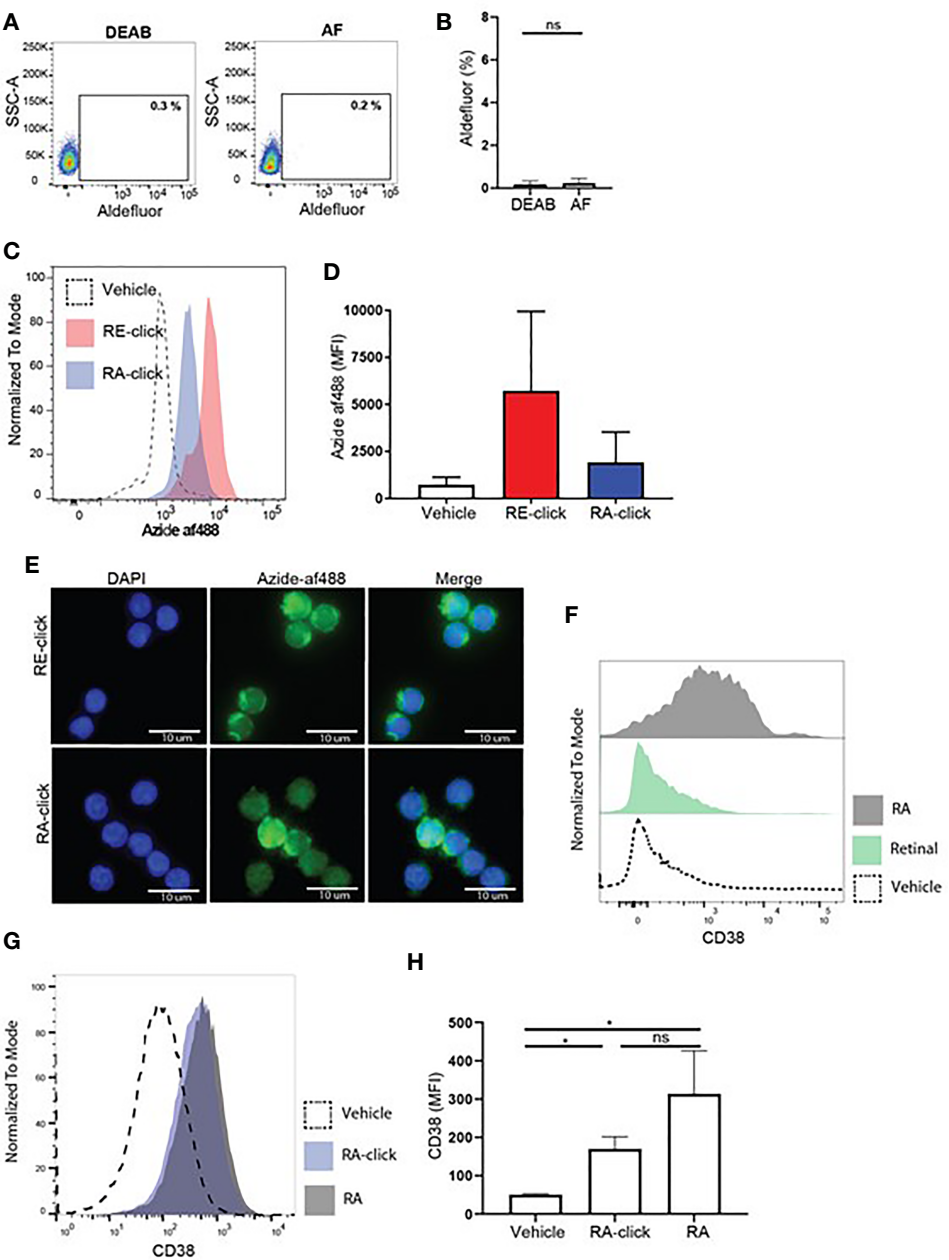
Human naive B cells cannot produce retinoic acid, but are affected by exogenous retinoids to upregulate CD38 expression. Human naive B cells were isolated from peripheral blood. **(A)** Representative dot plot of aldefluor positivity in naive B cells. DEAB reagent was used as a negative control. **(B)** Quantification of aldefluor positivity in naive B cells from 3 different donors. **(C)** Representative histogram of the uptake of RE-click (pink) and RA-dick (blue) in naive B cells compared to vehicle control (dotted line). **(D)** Quantification of the uptake of RE-click and RE-click in naive B cells compared to vehicle control from 3 different donors. **(E)** Visualization of the RE- and RA-click using Azide-AF488 (green) with a nucleus staining (DAPI, blue) in naive B lymphocytes from 5 different donors. **(F)** Human naive B cells were isolated from peripheral blood and cultured in the presence of T cell dependent (TD) stimuli for 7 days, including interleukin-4, anti-CD40 and anti-IgM antibodies. Histogram of CD38 expression on B cells upon addition of unmodified retinal (green) or retinoic acid {grey) in comparison to vehicle control (dotted line). **(G)** Histogram of CD38 expression upon culturing with RA-click (blue), its unmodified counterpart retinoic acid (grey) and vehicle control (dotted line) during 7 day TC-dependent stimulation. **(H)** Quantification of CD38 expression upon culturing with the RA-click, its unmodified counterpart retinoic acid and vehicle control from 4 different donors. Data are presented as mean ± SD. ANOVA; *p < 0.05. ns indicates p > 0.05.

After validating the clickable probes within human DCs, we assessed vitamin A uptake and functionality within the less well studied B lymphocyte compartment. Although it is recognized that RA-mediated processes in B cells are important for proper adaptive immune responses, such as IgA class-switching (14, 17, 19, 35, 36) and induction of CD38 (37, 38), the physiological mechanism remains unclear. To address the effects of retinoids on human B lymphocytes, we first used the aldefluor assay to test whether B lymphocytes have the machinery to produce retinoic acid. Within the total CD19^+^ B lymphocyte population present in peripheral blood, a small proportion was positive for the aldefluor assay compared to DEAB treated samples (**Supplemental Figures 3A, B**). However, when only naïve B cells were analysed no differences were observed in MFI between cell treated with vehicle or DEAB, demonstrating that human naïve B cells from peripheral blood cannot metabolize the aldefluor assay substrate. This result suggests that naïve B cells lack ALDH activity (**Figures 4A, B**).

Next, we addressed whether clickable retinoids could be made visible in B cells present in total PBMC fractions. Hereto, we first tested whether flow cytometry and click chemistry were compatible. We observed that the cupper-mediated click reaction influenced the forward and side scatter profile of leukocytes (**Supplemental Figure 3A**) as well as the intensity of multiple fluorescently labelled antibodies (**Supplemental Figures 3B–D**). This disqualified the combination of flow cytometry and click chemistry in one assay as a viable method for proper analysis of immune subsets. Therefore, we decided to first use magnetic bead isolation to obtain naïve B cells, followed by probe incubation and the click reaction, in order to address whether B lymphocytes can take up retinoid probes. The RE-probe accumulated with high MFI within the cells compared to the vehicle control, suggesting that naïve B cells efficiently took up the probe (**Figures 4C, D**). The RA-click was taken up as well, however the MFI was lower when compared to RE-click. Similarly, total CD19^+^ B cells, including naïve as well as memory B cells, showed a higher MFI with RE-click when compared to RA-click (**Supplemental Figures 4C, D**). Next, we examined the intracellular location of the accumulated retinoid probes. Hereto, naïve B cells were placed on microscope slides upon probe incubation and analyzed microscopically. Human naïve B cells treated with vehicle control demonstrated low fluorescence intensity, whereas incubation of the RE- and RA-click showed increased fluorescent signal (**Supplemental Figure 5**). The RE-click probe preferably located outside the nucleus (**Figure 4E**). The RA-click was distributed evenly in most naïve B cells, while some cells showed also accumulation of RA-click inside the nucleus (**Figure 4E**). The number of cells with intra-nuclear probe accumulation varied per donor. To examine whether the probes were taken up by the cells, rather than non-specific binding to the outer cell membrane, three dimensional analysis was performed. Here, we found that both the RE- as the RA-click were inside the cells, and had crossed the cell membrane (**Supplemental Videos 3** and **4**).

Next, biological functionality of the clickable probes was tested within naïve B lymphocytes. Hereto we first addressed whether culture of human naïve B cells with T cell dependent stimuli including αIgM antibodies, αCD40 antibodies and interleukin-4 with the natural form of retinal or RA for 7 days could affect CD38 expression. We observed that natural RA induced upregulation of CD38 on naïve B lymphocytes, while its substrate retinal had no effect (**Figure 4F**). In line, we observed that both RA-click as well as natural RA induced CD38 expression on naïve B cell in a similar fashion (**Figures 4G, H**). For the induction of IgA on the cultured B cells in these conditions we observed that both RA-click, as well as its natural equivalent, increased the percentage and fluorescent intensity of IgA on naïve B lymphocytes, however this was not significant (**Supplemental Figure 6**). Taken together our data suggest that, despite lacking ALDH activity, retinal and retinoic acid are taken up by naïve B cells, and that RA induces CD38 expression *in vitro*.

### Monocyte-Derived Dendritic Cells Convert Retinal Into Retinoic Acid, and Increase B Cell CD38 Expression

**Figure 5:**
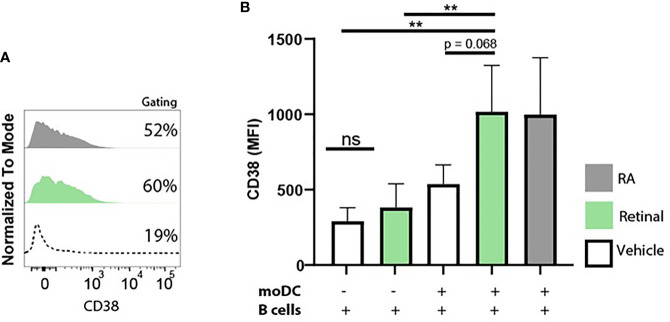
Monocyte-derived dendritic cells convert retinal into retinoic acid, and increase B cell CD38 expression. Naive B cells and monocyte-derived dendritic cells were co-cultured in vitamin A deficient medium in the presence of TD-dependent stimuli for 9 days, including interleukin-4, anti-CD40 and anti-IgM antibodies. As a negative control naive B cells and monocyte-derived dendritic cells were co-cultured with vehicle alone. **(A)** Representative histogram of CD38 expression on naive B cells supplemented with natural retinal (green) or RA (black) during the 9 day coculture. Vehicle treatment is indicated with the dotted line. As a negative control, co­cultures were treated with vehicle alone (dotted line). **(B)** Quantification CD38 mean fluorescent intensity of naive B cells supplemented with unmodified retinal (green), RA (black) or vehicle treated cells (white) from 4 separate experiments with individual blood donors (n=4). Data are presented as mean ± SD. ANOVA; **p < 0.01.

Since we observed that moDCs have the capacity to take up and convert retinal into retinoic acid, we further addressed whether dendritic cells can serve as a source of retinoic acid for naïve B cells *in vitro* using the upregulation of CD38 as a read-out. To this end, we set up a co-culture of naive B lymphocytes with moDCs and T cell dependent stimuli including αIgM antibodies, αCD40 antibodies and interleukin-4 with for 9 days. These co-cultures were carried out in Vitamin A deficient medium as regular FCS-supplemented medium contains retinal, which allowed us to analyse the effect of the addition of retinal in the absence or presence of moDCs on B cells. As a positive control, RA was supplemented to the moDC and B cell co-culture leading to increased expression of CD38 (**Figures 5A, B**). Addition of retinal resulted in enhanced CD38 expression only when moDCs and naïve B cells were cultured together and not when naïve B cells were cultured alone(**Figures 5A, B**). Overall, this data indicates that human dendritic cells can convert retinal into retinoic acid, which can be transferred to naïve B cells, leading to B cell differentiation.

## Discussion

In this study, we designed clickable probes which closely resemble the natural homologues, allowing analysis of the retinoid uptake within human immune cells. A protocol was established to study retinoid probes in a wide variety biological analysis systems, including flow cytometry and microscopy. As a result, we observed that multiple immune cell populations including monocytes, granulocytes, dendritic cells and B lymphocytes take up retinoids, to varying degrees of intensity, indicating that these probes address cell specific uptake of vitamin A metabolites by immune cells. Human dendritic cells and B lymphocytes were both able to internalize the retinoid analogues. Although dendritic cells have the machinery to convert retinal into retinoic acid, our data shows that B lymphocytes do not express ALDH enzymes, which is in line with earlier reports (39). Despite the incapability of B cells to produce RA, IgA class switching by B lymphocytes is facilitated by RA (11, 35). In addition to existing data, we now demonstrate that human dendritic cells convert retinal and provide B lymphocytes with retinoic acid resulting in the upregulation of CD38 expression. This suggests that similar as has been shown with mouse studies, also for human B lymphocytes RA steers adaptive immune responses and that DCs can serve as an exogenous source of RA to facilitate this process.

The visualization of lipid mediators at cellular levels has been a challenge. Although mass spectrometry imaging protocols have been optimized to detect small lipid mediators within cell structures (40, 41), this requires specialized equipment, specialized knowledge and is time consuming. To overcome this problem, researchers have used the chemical process of copper(I)-catalyzed azide-alkyne cycloaddition to a variety of lipid structures including fatty acids and cholesterols (42–45). Previously, we developed an activity-based probe with alkyne click handle to visualize and quantify ALDH activity (27). In line, we now succeeded in chemically adjusting retinal and retinoid acid, enabling their visualization in flow cytometry and microscopy approaches and showing similar functionality compared to their natural counterpart. Nevertheless, the retinoid probes have two practical limitations, which we did not further elaborate on during this study. We observed that concentrations between 100 – 1000 nM of retinoid probes in moDCs result in significant uptake, while this was not obvious at the concentration 1 – 10 nM, during an incubation time of 1 hour. To quantify physiological relevant concentrations of our probes, longer incubation times might be required. However, upon longer incubation with the probes at 37°C, we observed that the probes were metabolized as the fluorescence intensity signal decreased when incubation time was extended. Yet, when incubated at 4°C the uptake of the probes increased when cells were incubated for 4 hours, compared to 1 hour. This is in line with a report stating that therapeutically administered retinoic acid is metabolized in patients in a period of 30-40 min (46). Moreover, we experienced that the chemical clicking process of the fluorescent label affected fluorescent intensity of some commercially available antibodies, making it difficult to combine retinoid visualization with additional markers. To circumvent this, immune populations were isolated using magnetic beads or flow cytometry sorting prior to the chemical clicking method, to allow assessment of uptake within specific subsets without using additional fluorescent antibodies. However, copper-mediated click chemistry has been successfully combined with immunofluorescence by others (47, 48). One study demonstrated that specific fluorophores are copper-resistant, such as td Tomato, allowing to combine conventional staining with copper-mediated chemistry (47). Consequently, additional studies are required to determine which fluorescently labelled antibodies can withstand copper-mediated chemistry to combine click-chemistry with flow cytometry in future studies.

It is uncertain whether retinoids passively diffuse into cells because of their hydrophobic properties, or whether it is a process that requires specific receptors or transporters. Transport of the vitamin A derivative retinol in the periphery takes place when coupled to serum transport proteins, such as retinol binding protein 4 (RBP4) (31), and uptake is facilitated by a receptor expressed on target cells (31). We questioned whether the uptake of our clickable probes in moDCs, required any carrier proteins. However, we observed that the probes were still efficiently internalized when the incubations were performed with PBS buffer lacking any further supplementation, meaning that the clickable retinoids had no access to any transport proteins. This suggests that clickable probes were not internalized *via* transport proteins. Moreover, it is also unlikely that clickable retinoids entered immune cells in our experiments *via* the retinoic acid 6 (STRA6) receptor, which has been described to be involved in retinol uptake, as unbound retinoids cannot be transported *via* STRA6 (49, 50). Others have proposed that uptake of free retinoids is reliant on passive diffusion facilitated by their size and hydrophobic properties (32–34). However, passive diffusion would be an inconvenient mechanism as free retinoids are highly toxic to cells (51, 52). Furthermore, we demonstrated that both the RE- and RA-click probes can efficiently be taken up when incubated on ice, suggesting that no active metabolism is required for the internalization. Although it may not be an ATP-dependent mechanism, we did observe that only half of the moDCS internalized RA-click, suggesting that the uptake of retinoids is not based purely on passive diffusion but rather on other mechanism such as facilitated diffusion or by binding to specific receptors. The clickable retinoid probes will allow to further study this mechanism at more detail.

We demonstrated that human peripheral B lymphocytes do not possess ALDH enzymatic activity, but rely on DCs for RA production. In the human gut, CD103^+^ ALDH^+^ DCs are residing within the lamina propria near the epithelium (53, 54). It was demonstrated that human epithelial cells induce tolerogenic properties in monocyte-derived DCs *in vitro (* 24). Moreover, mouse studies demonstrated that insufficient development of LP tolerogenic DCs due to insufficient vitamin A metabolism, leads to an impaired capacity to facilitate mucosal IgA class switching (11, 12, 55, 56). The effect of RA for IgA expression within human B cells has been demonstrated by others (57). Yet, within this study neither our probes, nor natural RA, were able to significantly induce IgA class switching within B lymphocytes *in vitro*. This might be due to slightly different variations in the protocols, which we did not further explore. Tolerogenic DCs are migratory cells and have been observed to travel from the LP into the MLNs using CCR7 (58). It was reported in mice that these LP-derived DCs influence the expression of the gut homing receptor α4β7 on T cells within the MLNs, using retinoic acid (9, 58–60). Similarly, tolerogenic DCs reside within human MLNs and have the ability to induce α4β7 expression on T cells (10, 24). Therefore, we postulate that human tolerogenic DCs produce RA and are involved in the induction of IgA class switching locally within the LP, but also in mucosal lymphoid tissues such as the MLNs.

Thus, in this study, we provide user friendly clickable retinoid probes, which can be used to visualize and examine functionality of retinoids in a wide variety biological systems. We demonstrate that multiple human immune populations have the capacity to internalize retinoids to varying degrees. Naïve B lymphocytes cannot metabolize retinoids themselves because they lack ALDH1 activity, and thus rely on additional cells for RA production. We showed that human DCs provide RA to B cells, leading to upregulation of CD38. Together with existing data based on mouse studies, these observations support that human DCs facilitate adaptive B cell immunity in a retinoic acid dependent manner. Taken together, our data provide valuable insight into the mechanism behind vitamin A metabolism and the importance of retinoids for human adaptive immunity.

## Data Availability Statement

The raw data supporting the conclusions of this article will be made available by the authors, without undue reservation.

## Author Contributions

AB wrote the manuscript, performed experiments and analysed the data. MNE performed experiments. SK designed, synthesized and validated the retinoid probes. MS, ME, and RM supervised the experiments and analyses. All authors contributed to the article and approved the submitted version.

## Funding

This project received funding from the Institute for Chemical Immunology (ICI) and Oncode Institute.

## Conflict of Interest

The authors declare that the research was conducted in the absence of any commercial or financial relationships that could be construed as a potential conflict of interest.
