# Supplementary material for: Clickable Vitamins as a New Tool to Track Vitamin A and Retinoic Acid in Immune Cells
Source: Front Immunol. 2021 Jul 8;12:671283. doi: 10.3389/fimmu.2021.671283 (PMC8298001; doi:10.3389/fimmu.2021.671283)
Supplement: Supplementary file 1 [file Presentation_1.pptx]

## Slide 1
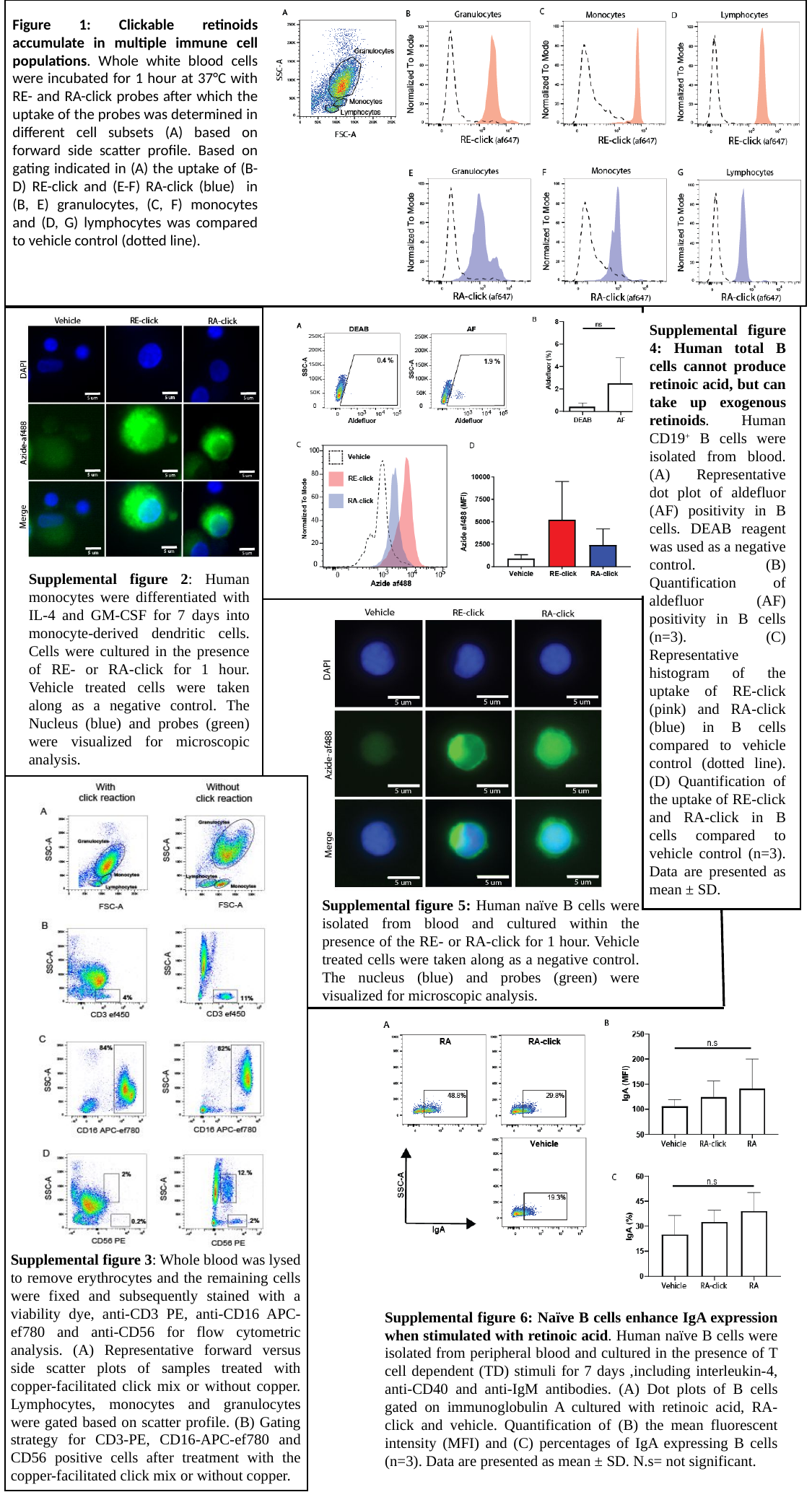

Figure 1: Clickable retinoids accumulate in multiple immune cell populations. Whole white blood cells were incubated for 1 hour at 37°C with RE- and RA-click probes after which the uptake of the probes was determined in different cell subsets (A) based on forward side scatter profile. Based on gating indicated in (A) the uptake of (B-D) RE-click and (E-F) RA-click (blue) in (B, E) granulocytes, (C, F) monocytes and (D, G) lymphocytes was compared to vehicle control (dotted line).
Supplemental figure 4: Human total B cells cannot produce retinoic acid, but can take up exogenous retinoids. Human CD19+ B cells were isolated from blood. (A) Representative dot plot of aldefluor (AF) positivity in B cells. DEAB reagent was used as a negative control. (B) Quantification of aldefluor (AF) positivity in B cells (n=3). (C) Representative histogram of the uptake of RE-click (pink) and RA-click (blue) in B cells compared to vehicle control (dotted line). (D) Quantification of the uptake of RE-click and RA-click in B cells compared to vehicle control (n=3). Data are presented as mean ± SD.
Supplemental figure 2: Human monocytes were differentiated with IL-4 and GM-CSF for 7 days into monocyte-derived dendritic cells. Cells were cultured in the presence of RE- or RA-click for 1 hour. Vehicle treated cells were taken along as a negative control. The Nucleus (blue) and probes (green) were visualized for microscopic analysis.
Supplemental figure 5: Human naïve B cells were isolated from blood and cultured within the presence of the RE- or RA-click for 1 hour. Vehicle treated cells were taken along as a negative control. The nucleus (blue) and probes (green) were visualized for microscopic analysis.
Supplemental figure 3: Whole blood was lysed to remove erythrocytes and the remaining cells were fixed and subsequently stained with a viability dye, anti-CD3 PE, anti-CD16 APC-ef780 and anti-CD56 for flow cytometric analysis. (A) Representative forward versus side scatter plots of samples treated with copper-facilitated click mix or without copper. Lymphocytes, monocytes and granulocytes were gated based on scatter profile. (B) Gating strategy for CD3-PE, CD16-APC-ef780 and CD56 positive cells after treatment with the copper-facilitated click mix or without copper.
Supplemental figure 6: Naïve B cells enhance IgA expression when stimulated with retinoic acid. Human naïve B cells were isolated from peripheral blood and cultured in the presence of T cell dependent (TD) stimuli for 7 days ,including interleukin-4, anti-CD40 and anti-IgM antibodies. (A) Dot plots of B cells gated on immunoglobulin A cultured with retinoic acid, RA-click and vehicle. Quantification of (B) the mean fluorescent intensity (MFI) and (C) percentages of IgA expressing B cells (n=3). Data are presented as mean ± SD. N.s= not significant.
